# Genome-wide characterization of the wheat cryptochrome/photolyase family identifies TaCRY1b as a lamina-joint-associated protein interacting with TaLG2 and TaLG2L

**DOI:** 10.3389/fpls.2026.1905632

**Published:** 2026-08-14

**Authors:** Bingyan Gu, Yimo Wang, Chang Liu, Ziyu Niu, Jiaxin Liu, HaiZheng Wang, Yifan Zhang, Chunyang Wang, Rongna Wang

**Affiliations:** 1 State Key Laboratory of North China Crop Improvement and Regulation/Key Laboratory of Hebei Province for Plant Physiology and Molecular Pathology/College of Life Sciences, Hebei Agricultural University, Baoding, China; 2 School of Health Care and Medicine, Zibo Polytechnic University, Zibo, China

**Keywords:** cryptochrome, leaf angle, TaCRY1, TaLiguless2, wheat

## Abstract

**Background:**

Cryptochromes (CRYs) are blue light and ultraviolet A (UV-A) photoreceptors that play pivotal roles in regulating plant development and stress responses through mediating light signaling pathways. However, the evolutionary and functional characteristics of the cryptochrome/photolyase (*CRY/PHL*) family in wheat (*Triticum aestivum* L.) remain poorly understood.

**Results:**

57 *CRY/PHL* genes were identified across eight species, including 14 members in hexaploid wheat. Phylogenetic analysis classified *CRY/PHL*s into four subfamilies. All TaCRY/PHL proteins contain the conserved photolyase homology region (PHR), whereas the cryptochrome C-terminal (CCT) domain is exclusively present in the CRY1 subfamilies. Collinearity analysis revealed extensive synteny among wheat, rice, and maize *CRY/PHL*s, but not with *Arabidopsis*, indicating lineage-specific evolution within grasses. Promoters of *TaCRY/PHL* genes contain multiple predicted light-, hormone-, and stress-responsive cis-elements. Tissue-specific expression profiling demonstrated that subfamily A and C members are predominantly expressed in vegetative tissues, while subfamily B and D members are mainly expressed in reproductive organs. Notably, *TaCRY1b* expression progressively increased during leaf lamina joint (LJ) development. Subcellular localization demonstrated that TaCRY1b localizes to both the nucleus and cytoplasm, while bimolecular fluorescence complementation assays uncovered direct physical interactions between TaCRY1b and TaLiguless2 (TaLG2s), key regulators of LJ formation. AlphaFold3 prediction showed a putative interaction interface between TaLG2/TaLG2L and TaCRY1b, where multiple complementary residue pairs form polar contacts predominantly within the PHR domain of TaCRY1b.

**Conclusions:**

This study provides a comprehensive framework for the evolution and functional diversification of the wheat CRY/PHL family and identifies TaCRY1b as a promising candidate regulator of lamina joint development, laying a solid foundation for further investigations into its moltcular function in leaf angle modulation.

## 1 Introduction

Cryptochromes (CRYs) are blue light and ultraviolet A (UV-A) receptors widely distributed across fungi, algae, land plants, and animals, which play crucial roles in various physiological processes (Wang et al., 2016). In plants, CRYs regulate various aspects of plant life cycles through blue light-mediated gene expression, including seedling de-etiolation (Ren et al., 2024), photoperiodic flowering (Lin et al., 1996), circadian rhythms (Wang et al., 2021), hypocotyl elongation (Ma et al., 2018), cotyledon expansion (Daniele et al., 2006), stomatal development (Kang et al., 2009), leaf senescence (Meng et al., 2013), and flavonoid biosynthesis (Hao et al., 2025). Beyond photomorphogenesis, CRYs also participate in abiotic and biotic stress responses. For instance, *AtCRY1* enhances immunity against pathogens (Hao et al., 2025). Heterologous expression of wheat *TaCRY1a* and *TaCRY2* in Arabidopsis increases salt sensitivity (Xu et al., 2009). Suppression of rice *CRY1b* reduces melatonin and brassinosteroid biosynthesis, indirectly improving salt tolerance (Hwang and Back, 2021).

Cryptochromes were first discovered in *Arabidopsis thaliana*, where three *CRY* subfamilies were identified, including *CRY1*, *CRY2*, and *CRY-DASH (CRY3)* (Ma et al., 2018). However, the composition and copy number of *CRY* subfamilies vary considerably across species. For instance, the moss *Physcomitrella patens* contains two *CRY* members (Sun et al., 2006), while the fern *Adiantum capillus-veneris* contains five (Imaizumi et al., 2000). To date, systematic analyses of *CRY* genes in angiosperms have been reported for only a few dicot and monocot species. Most plant cryptochromes are flavoproteins with molecular masses ranging from 70 to 80 kDa and possess two conserved functional domains, including the N-terminal photolyase homology region (PHR) and the cryptochrome C-terminal (CCT) domain (Shao et al., 2020). Upon blue light absorption, the highly conserved PHR domain, which non-covalently binds flavin adenine dinucleotide (FAD), triggers FAD photoreduction, leading to conformational changes that activate CRY photoreceptor activity via protein dimerization or polymerization and thus directly mediate blue light signal transduction (Klar et al., 2007; Wang et al., 2021; Wang and Lin, 2025). The CCT domain mediates direct interaction of CRY proteins with COP1, leading to dissociation of the COP1/SPA complex and promoting accumulation of HY5 and CO transcription factors, which ultimately advance photomorphogenesis and flowering (Wang et al., 2021; Liu et al., 2025).

Wheat (*Triticum aestivum* L.) is a major staple crop worldwide. Photoperiod and light quality directly influence its heading date, tiller number, and grain filling (Boden et al., 2015; Dreccer et al., 2022). As blue light receptors, CRY proteins sense changes in light intensity and photoperiod, regulating the CO-FT pathway, the circadian clock, and photomorphogenesis, thereby determining flowering time and yield potential (Liu et al., 2018; Wang et al., 2021). Plant architecture traits, including plant height, leaf shape, leaf angle, and spike morphology, significantly affect yield (Li et al., 2023). Leaf angle, defined as the angle between the leaf midrib and the stem, is a critical determinant of canopy light interception and photosynthetic efficiency (Zhai et al; Tian et al., 2024). Erect leaves reduce self-shading, improving light distribution and enabling higher planting densities, which ultimately increases grain yield (Liu et al., 2019; Tian et al., 2019; Wang et al., 2020). Photoreceptors sense dynamic changes in environmental light and directly interact with transcription factors to regulate leaf angle under varying densities and conditions. For example, in maize, phytochrome B and LIGULELESS1 (LG1) regulate leaf angle by modulating *ZmHB53* transcription (Shi et al., 2024). In *Arabidopsis*, *CRY1* and *CRY2* participate in gibberellin (GA) signaling (Zhao et al., 2020; Zhong et al., 2021). Moreover, GA were reported to modulate plant architecture by interacting either synergistically or antagonistically with brassinosteroids in rice (Tong et al., 2014; Tang et al., 2017). Wheat TaCRY1a interacts with the GA receptor TaGID1 and DELLA proteins via its C-terminal domain in a blue light-dependent manner, competitively inhibiting GID1-DELLA binding and stabilizing DELLA proteins, which represses stem elongation and affects plant height (Yan et al., 2021). However, whether TaCRY/PHL are associated with leaf angle in wheat remains unclear.

Understanding the evolutionary history of the *CRY/PHL* family in wheat and its wild relatives is essential for functional characterization and breeding applications. In this study, we systematically identified *CRY/PHL* genes in five wheat species representing different ploidy levels and evolutionary lineages, including *T. urartu* (AA), *Ae. tauschii* (DD), *T. turgidum* (AABB), *T. dicoccoides* (AABB), and *T. aestivum* (AABBDD). A total of 43 *CRY/PHL* genes were identified across these five species. Comprehensive analyses of phylogenetic relationships, gene structures, conserved motifs, synteny, cis-acting elements, and expression patterns were performed. Using transcriptomic data and qRT-PCR, we examined the expression profiles of *TaCRY/PHL* genes in different tissues and during leaf lamina joint development. Taken together, this study provides a comprehensive overview of the evolutionary divergence and functional specialization of wheat cryptochrome/photolyase members, and identified pivotal genes for future mechanistic dissection and molecular breeding.

## 2 Materials and methods

### 2.1 Plant materials and growth conditions

Wheat (*Triticum aestivum* L.) cultivar Chinese Spring seeds were germinated for 1 day and then sown in pots. Plants were grown in a controlled environment chamber at 20 °C with a photoperiod of 16 h light/8 h dark. The lamina joint, together with approximately 0.5 cm of adjacent leaf sheath and leaf blade tissues, was collected at 4 (Stage 1, S1), 8 (Stage 2, S2), and 12 (Stage 3, S3) days after germination. All tissues were sampled and immediately frozen in liquid nitrogen, and stored at –80 °C. Three independent biological replicates were collected for each time point and tissue.

### 2.2 Identification of cryptochrome/photolyase family genes in multiple wheat species

Downloaded the complete genome sequence, protein sequences, and gene structure annotation files for *Triticum aestivum*, *Triticum urartu*, *Triticum turgidum*, *Triticum dicoccoides* and *Aegilops tauschii* from the Ensembl Plants website (https://plants.ensembl.org/index.html). The protein sequences of wheat species were used as the database for screening in the experiment. Using the WheatOmics website, screen for relevant conserved domain data related to the wheat *CRY/PHL*. Hidden Markov Model (HMMs) for the conserved domains of the *CRY/PHL* family—DNA photolyase (PF00875) and FAD binding domain of DNA photolyase (PF03441)—were downloaded from the Pfam database (http://pfam.xfam.org/). Using the hmmersearch function in HMMER 3.0, these HMMs were searched against the protein sequences with an e-value<10–^5^ to ensure reliability. Candidate CRY/PHL proteins were identified as those that contained both domains. Finally, the presence of both CRY/PHL family domains in the candidates was validated using the InterProScan website (https://www.ebi.ac.uk/interpro/about/interproscan/). Chromosomal localization of wheat *CRY/PHL* genes was determined using the Gene Location Visualize from GTF/GFF tool in Tbtools software, with gene names assigned based on their chromosomal positions.

### 2.3 Phylogenetic distribution and evolutionary characteristics of *CRY/PHL*s in the plant kingdom

To investigate the evolutionary distribution of the *CRY/PHL* family across the plant kingdom, a comprehensive phylogenetic analysis was performed using 73 species covering major green plant lineages (**Supplementary Table 1**). Firstly, the protein sequences were compared using the MAFFT software, and then the sequences were trimmed using the trimAl software to remove low-quality regions. During the tree-building stage, the IQ-TREE 2 software was used to construct the maximum likelihood evolutionary tree, and the iTOL platform was utilized to visualize the evolutionary tree. Finally, based on the screening criteria of Bootstrap value ≥ 70 and the number of branch genes ≥ 3, a total of 12 major evolutionary clades were identified.

### 2.4 Physicochemical characterization of the cryptochrome/photolyase family

Use the online software Expasy ProtParam (https://web.expasy.org/protparam/) to analyze the physicochemical properties of the CRY/PHL protein, including amino acid count, molecular weight, isoelectric point, instability index, fat index, and average hydrophilicity. Employ WoLF PSORT (https://wolfpsort.hgc.jp) to predict the subcellular localization of the CRY/PHL protein.

### 2.5 Phylogenetic tree construction and synteny analysis of the cryptochrome/photolyase family

The *CRY/PHL* gene IDs for *Arabidopsis thaliana*, rice, and maize were obtained from the database TAIR (https://www.arabidopsis.org), CHINA RICE DATA CENTER (https://www.ricedata.cn), and MaizeGDB (https://maizegdb.org), respectively. Protein sequences of *CRY/PHL* family for these species, *Triticum urartu*, *Triticum turgidum*, *Triticum dicoccoides* and *Aegilops tauschii* and *Triticum aestivum* were collected and aligned using MAFFT v7.526. Low-confidence regions in the alignment were removed with TrimAl v1.5.rev0. A maximum likelihood phylogenetic trees were then constructed using IQ-TREE v2.3.6 with 1, 000 bootstrap replicates for branch support. Finally, the phylogenetic tree was visualized using the iTOL website (https://itol.embl.de). Synteny analysis was performed among the genomes of wheat, rice, and maize using the MCScanX tool in TBtools. The results were visualized to examine syntenic relationships across the three species.

### 2.6 Structural analysis of *CRY/PHL* family genes, conserved motifs and domains

Conserved motifs within CRY/PHL proteins were identified using the MEME Suite web server (https://meme-suite.org/meme/). The analysis was configured with the following parameters: a maximum of 10 predicted motifs and a width range from 6 to 50 amino acids. To identify conserved domains in the CRY/PHL proteins, the sequences were submitted to the NCBI Batch CD-Search website. The results, along with *CRY/PHL* gene structural annotations, were visualized using the Gene Structure View feature in Tbtools.

### 2.7 Analysis of cis-acting elements in the cryptochrome/photolyase family

The 2000 bp sequences immediately upstream of the translation start codon (ATG) of each *TaCRY/PHL* gene were extracted using the Gtf/Gff3 Sequence Extract function in TBtools. These sequences were submitted to the PlantCARE website (https://bioinformatics.psb.ugent.be/webtools/plantcare/html/) for prediction of promoter cis-acting element. The identified cis-regulatory elements were then visualized visualized using the ggplot2 package in R.

### 2.8 Analysis of expression patterns in the wheat cryptochrome/photolyase family

To explore the tissue-specific expression patterns of *TaCRY/PHL* genes, we utilized a transcriptomic dataset of 201 wheat samples that had been uniformly processed and analyzed in our previous study (Liu et al., 2026). The mean FPKM values of each *TaCRY/PHL* member were calculated for each tissue group, scaled using the scale() function, and visualized using the ComplexHeatmap package in R. Because the analysis combines samples from multiple studies without explicit batch-effect correction, these expression profiles should be interpreted as exploratory and descriptive (Liu et al., 2026).

### 2.9 RNA extraction and qRT-PCR

Total RNA was isolated from tissues with three biological replicates using using the Eastep^®^ Super Total RNA Extraction Kit (Promega, USA). Subsequently, cDNA was generated using the PrimeScript™ RT Reagent Kit with gDNA Eraser (Takara, Beijing, China) and performed as a template for quantitative real-time PCR (qRT-PCR) analysis. qRT-PCR were conducted on an Archimed X6 Time-Resolved Quantitative PCR System using the TB Green^®^ Premix Ex Taq™ II (Takara, Beijing, China), following the manufacturer’s recommended protocol. Each sample was replicated three times. Relative expression levelswere normalized to the wheat glyceraldehyde-3-phosphate dehydrogenase gene (*TaGAPDH*) as the internal control using the 2^-ΔΔCt^ method. Primers used for qRT-PCR are listed in **Supplementary Table 2**.

### 2.10 Subcellular localization

The full-length coding sequence (CDS) of *TaCRY1b* was amplified and cloned into the pMDC43 vector to generate a C-terminal GFP fusion construct under the control of the CaMV 35S promoter. The construct (35S-GFP-TaCRY1b) and the empty vector (GFP control) were introduced into *Nicotiana benthamiana* leaves via *Agrobacterium*-mediated transient transformation. After 48 h of infiltration, leaf epidermal cells were examined using confocal laser scanning microscopy to detect GFP fluorescence, Nuclear localization was assessed through co-infiltration with p2300-35S-H2B-mCherry.

### 2.11 Bimolecular fuorescent complementation analysis

The cDNA fragments of *TaCRY1b* and *TaLG2s* were amplified and cloned into pXY104-cYFP and pXY106-nYFP. The vectors were transformed into *Agrobacterium tumefaciens* strain *GV3101* and infiltrated into *N. benthamiana* leaves. The empty cYFP and nYFP or nYFP-TaLG2L and cYFP-TaCRY1b vector combination was used as a negative control. Infiltrated plants were maintained at 25 °C with a 16 h light/8 h dark photoperiod for 48 h after 12 h dark incubation. YFP signals were detected by confocal microscopy (Zeiss, LSM900).

## 3 Results

### 3.1 Identification and phylogenetic analysis of *CRY/PHL* family

**Figure 1:**
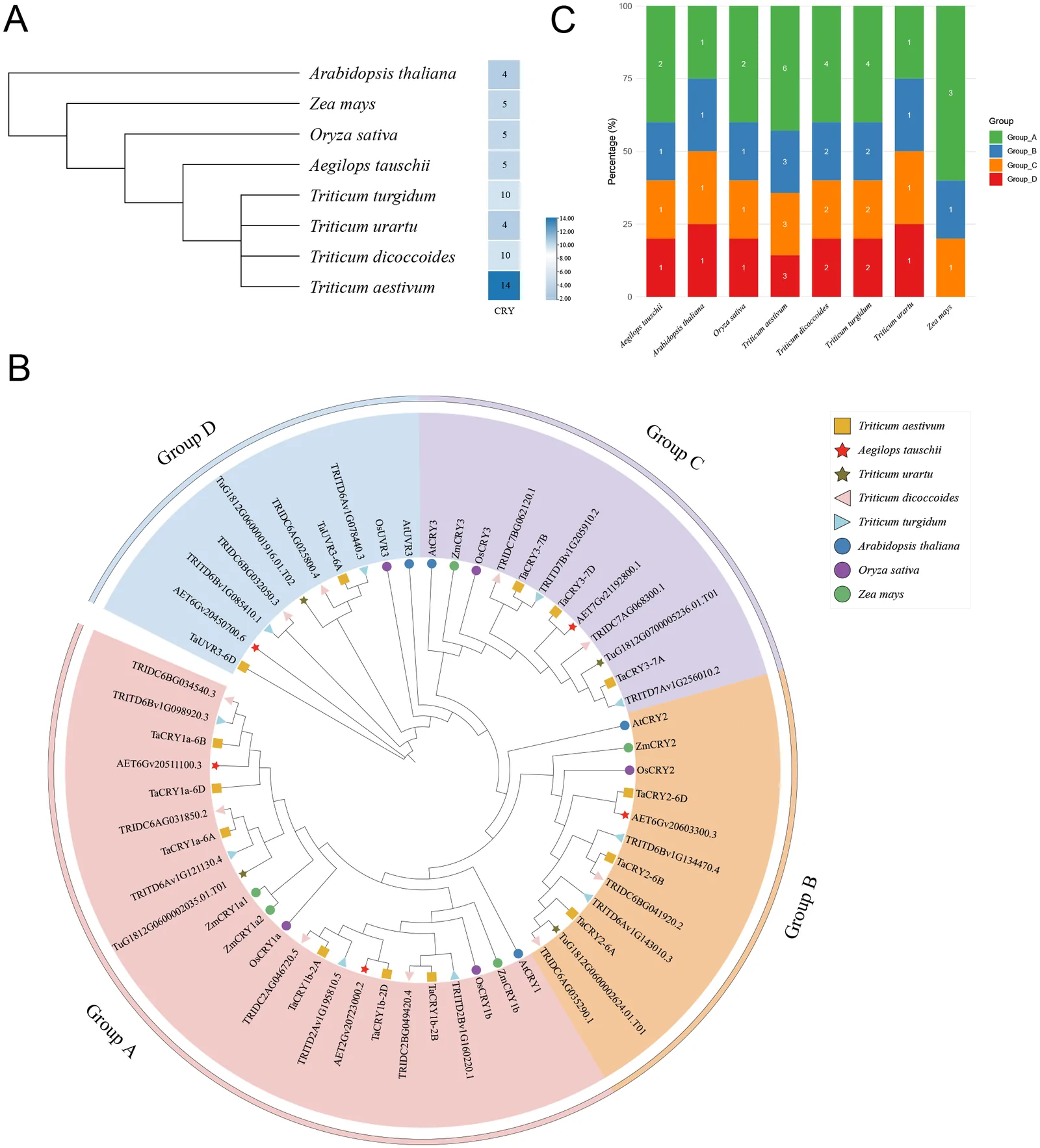
Phylogenetic analysis of *CRY/PHL*s in eight species. **(A)** Species evolution tree and the number of *CRY/PHL*s in eight species. **(B)** Phylogenetic analysis of *CRY/PHL*s in *Triticum aestivum* (Ta), *Triticum turgidum* (TRITD), *Triticum dicoccoides* (TRIDC), *Triticum urartu* (Tu), *Aegilops tauschii* (AET), *Oryza sativa* (Os), *Zea mays* (Zm), and *Arabidopsis thaliana* (At). **(C)** The number of different species in each of the four groups shown in **(B)**.

To elucidate the evolutionary relationships of CRY/PHL family members among common wheat and its ancestral species, as well as related model species, we conducted a comprehensive identification of *CRY/PHL* genes in eight species, including common wheat (*Triticum aestivum*, hexaploid, AABBDD), *Triticum urartu* (diploid, AA), *Triticum turgidum* (tetraploid, AABB), *Aegilops tauschii* (diploid, DD), *Triticum dicoccoides* (tetraploid, AABB), *Oryza sativa*, *Zea mays*, and *Arabidopsis thaliana*. A total of 57 *CRY/PHL* genes were identified (**Figure 1A**), including 14 in common wheat (**Supplementary Table 3**), 4 in *Triticum urartu*, 10 in *Triticum turgidum*, 5 in *Aegilops tauschii*, 10 in *Triticum dicoccoides*, 5 in *Oryza sativa*, 5 in *Zea mays*, and 4 in *Arabidopsis thaliana*. The fourteen CRY/PHLs in common wheat (*Triticum aestivum*, Ta) were named based on their homology with rice and *Arabidopsis thaliana* proteins (Matsumoto et al., 2003; Hirose et al., 2006; Chaves et al., 2011). A phylogenetic tree was constructed based on sequence alignment of CRY/PHLs in eight species and divided into four subgroups (**Figure 1B**). Group A comprises 23 CRY/PHLs, including *TaCRY1a-6A/6B/6D* and *TaCRY1b-2A/2B/2D*, showing highest sequence similarity to *OsCRY1a*, *OsCRY1b*, *ZmCRY1a1/a2*, *ZmCRY1b*, and *AtCRY1*. Within this group, proteins from *TaCRY1a* or *TaCRY1b* subgroups from A, B and D genomes share over 93% amino acid sequence identity. Group B comprises 12 CRY/PHLs, including *TaCRY2-6A/6B/6D*, which exhibit highest homology to *OsCRY2*, *ZmCRY2*, and *AtCRY2*. The paralogs between *TaCRY2* subgroups share over 95% amino acid sequence identity. Group C comprises 12 CRY/PHLs, including *TaCRY3-7A/7B/7D*, which show highest sequence similarity to *OsCRY3*, *ZmCRY3*, and *AtCRY3*. Proteins in *TaCRY3* subgroup of this group share over 91% amino acid sequence identity. Group D comprises 10 CRY/PHL members, including TaUVR3-6A/6D, showing high sequence similarity to OsUVR3 and AtUVR3. Additionally, the CRY/PHLs in the other four ancestral species of common wheat were present in all four Groups, with distribution ratios similar to those in bread wheat (**Figure 1C**), suggesting a high degree of conservation in the Group distribution patterns of CRY/PHLs between these relatives and bread wheat. Furthermore, all TaCRY/PHL clustered with their homologs from rice and maize on the same branch, reflecting the high sequence conservation of CRY/PHL proteins among wheat, rice, and maize.

### 3.2 Phylogenetic distribution and evolutionary characteristics of *CRY/PHL*s in the plant kingdom

**Figure 2:**
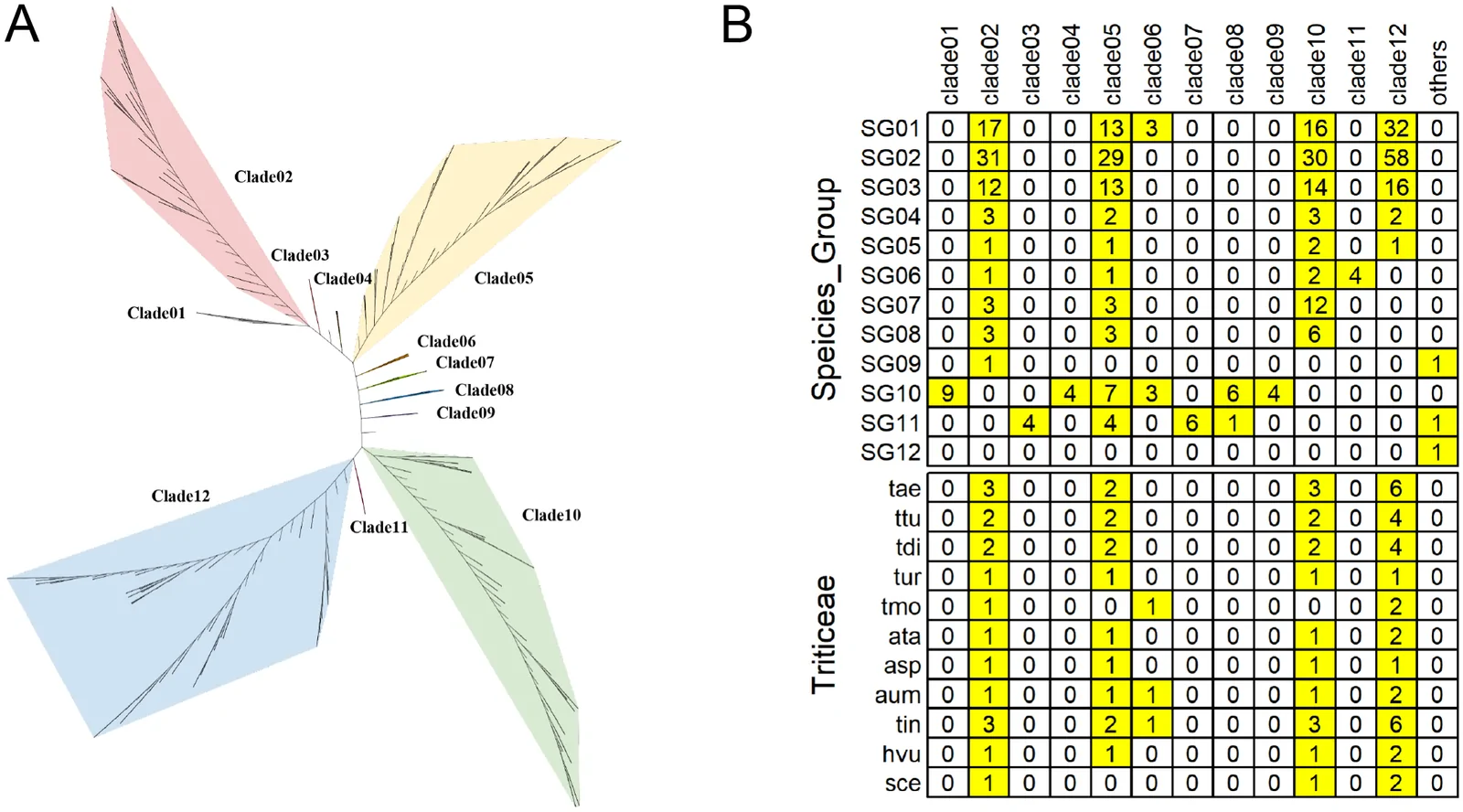
Phylogenetic distribution and evolutionary characteristics of *CRY/PHL*s in the Plant Kingdom. **(A)** Phylogenetic tree of *CRY/PHL*s in plants. **(B)** Distribution of CRY/PHLs across 12 groups (SG01-SG12). Triticeae (SG01), other Monocots (SG02), Eudicots (SG03), Angiosperms (not Monocots or Eudicots, SG04), Vascular plants (Gymnosperm, SG05), Vascular plants (Polypodiopsida, SG06), Vascular plants (Lycopodiopsida, SG07), Embryous plants (Bryophyta and Marchantiophyta, SG08), Streptophyta (SG09), Chlorophyte (SG10), Rhodophyta (SG11), and Bacteria (Cyanobacterium, as an outgroup, SG12). The Triticeae group includes *Triticum aestivum* (tae), *Triticum turgidum* (ttu), *Triticum dicoccoides* (tdi), *Triticum urartu* (tur), *Triticum monococcum* (tmo), *Aegilops tauschii* (ata), *Aegilops speltoides* (asp), *Aegilops umbellulata* (aum), *Thinopyrum intermedium* (tin), *Hordeum vulgare* (hvu), *Secale cereale* (sce).

*CRY*s were first discovered in *Arabidopsis thaliana* and represented one of the earliest evolved photoreceptors in plants (Chaves et al., 2011). To elucidate the phylogenetic distribution and evolutionary characteristics of CRY/PHLs across the plant kingdom, we constructed a phylogenetic tree based on 73 species covering a broad range of green plant lineages (Liu et al., 2026), including 36 monocots, 13 dicots, 3 basal angiosperms, 1 gymnosperm, 3 ferns, 3 bryophytes, 1 charophyte, 8 green algae, 4 red algae, and 1 cyanobacterium (as an outgroup, **Figure 2**). The results showed that the CRY/PHLs were divided into 12 clades (**Supplementary Table 1**; **Figure 2A**), including four major clades, designated as clade02 (72 members), clade05 (76 members), clade10 (85 members), and clade12 (109 members). According to the assignment of CRY/PHLs from *Arabidopsis*, rice, and wheat to the clades, the CRY/PHLs of all three species were found in the four major clades, where clade02, clade05, clade10, and clade12 corresponded to CRY3, UVR3, CRY2, and CRY1, respectively. Additionally, CRY/PHLs from other monocots, dicots, basal angiosperms, and gymnosperms were strictly distributed within the four major clades (clade02, clade05, clade10, and clade12) and were not found in any other clades (**Figure 2B**). Within the Triticeae species, all CRY/PHL members were also located in these four major clades except that *Thinopyrum intermedium*, *Triticum monococcum*, and *Aegilops umbellulata* each had one CRY/PHL member in clade06. Notably, clade06 also contained three members from the green algae, such as *Volvox carteri*, *Coccomyxa subellipsoidea*, and *Chlamydomonas reinhardtii*, suggesting that some Triticeae species may have retained a small number of ancestral clade members.

In *Ceratopteris richardii*, CRY/PHLs were distributed in clade02, clade05, clade10, and clade11 (**Figure 2B**), with clade11 clustering with clade12 but showing a bootstrap support value below 70%, suggesting that clade12 that corresponded to *CRY1* may have originated from an ancestor closely related to clade11. In more ancient lycophytes and bryophytes, CRY/PHLs were only distributed in clade02, clade05, and clade10. In lower algae, CRY/PHL members were mainly found in clade05 and several other small clades (**Figure 2B**). Among these, green algae possessed CRY/PHL members in clade01, which was closely related to clade02, suggesting the evolutionary origin of *CRY3* in higher plant. Other CRY/PHLs in green algal were also distributed in clade04, clade06, clade08, and clade09. Red algal CRY/PHLs were found in clade03, clade05, clade07, and clade08. Notably, the *Chara braunii* contained only two members, one was located in clade02, and the other formed a large branch (bootstrap = 100%) together with the clades containing *CRY2* and *CRY1*, implying the evolutionary origin of *CRY2* and *CRY1* may have emerged relatively late.

### 3.3 Chromosomal distribution and synteny analysis of wheat TaCRY/PHL members

**Figure 3:**
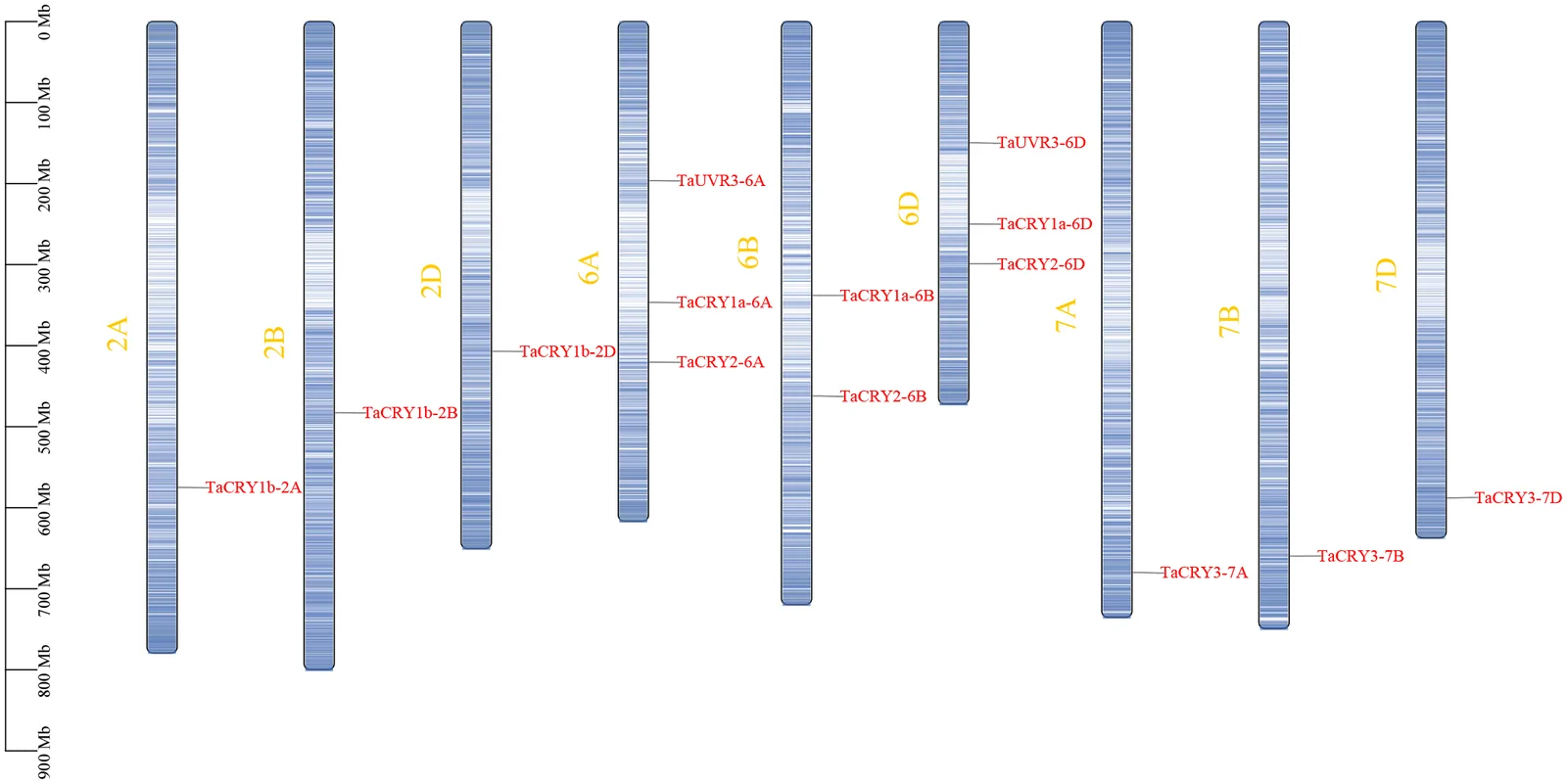
Distribution of *TaCRY/PHLs* on wheat chromosomes. The chromosome scale in millions of bases (Mb) is shown on the left.

**Figure 4:**
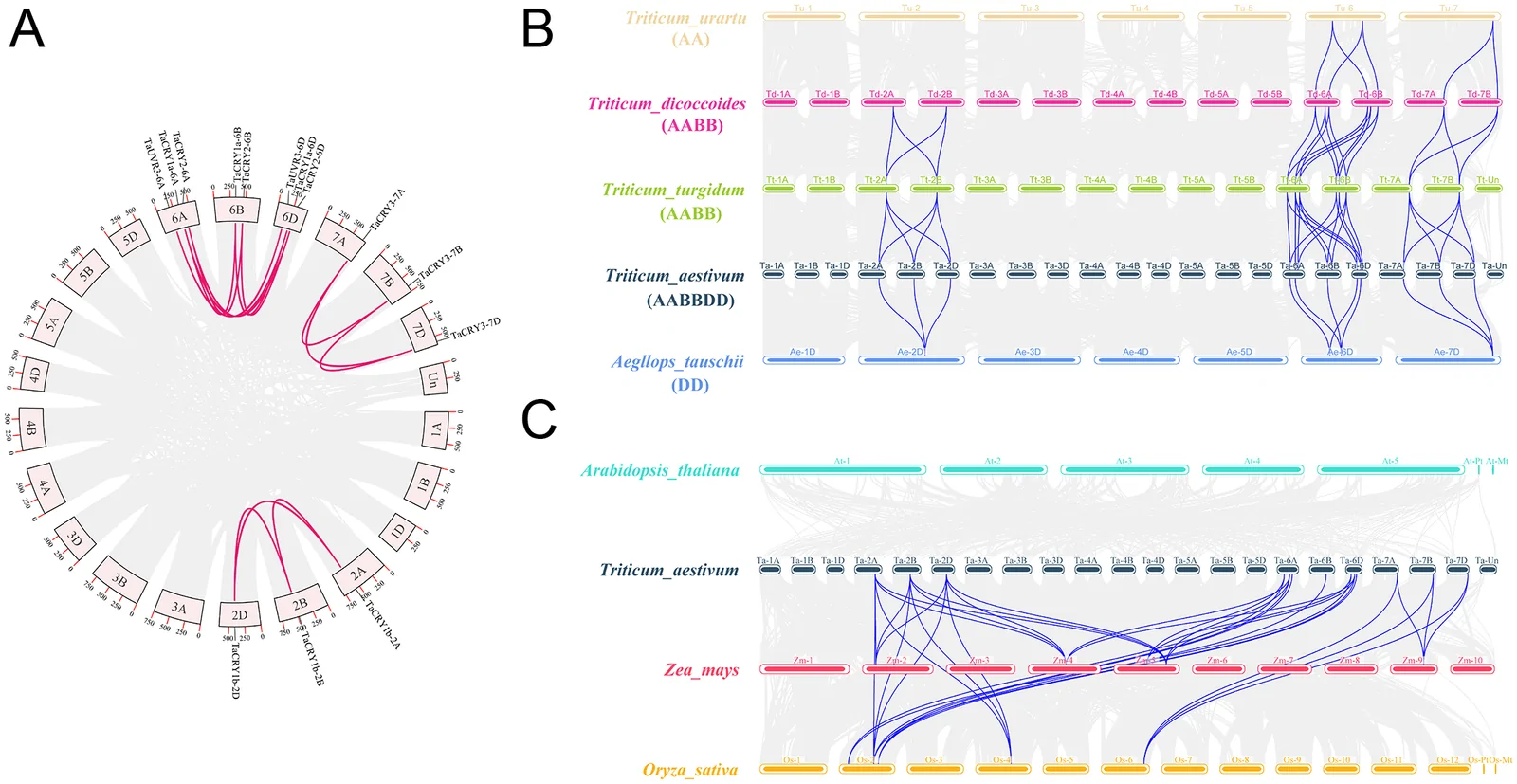
Synteny analysis of wheat *CRY/PHL* members. **(A)** Collinearity analysis of *TaCRY/PHL*s within *Triticum aestivum* (Ta). **(B)** Collinearity analysis of *CRY/PHL*s among *Triticum aestivum*, *Triticum turgidum*, *Triticum dicoccoides*, *Triticum urartu*, *Aegilops tauschii*. **(C)** Collinearity analysis of *CRY/PHL*s among *Triticum aestivum*, *Oryza sativa* and *Zea mays*.

The 14 *CRY/PHL* genes were mapped across nine chromosomes of common wheat, including 2A, 2B, 2D, 6A, 6B, 6D, 7A, 7B, and 7D (**Figure 3**). Among them, chromosomes 6A and 6D each harbor three members, chromosome 6B contains two, and remaining six chromosomes each possess one. To investigate the evolutionary relationships of the wheat CRY/PHL family, comprehensive synteny analyses were performed at both intraspecific and interspecific levels (**Figure 4**). Intraspecific synteny analysis revealed a total of 13 duplicated *TaCRY/PHL* gene pairs within the wheat genome (**Figure 4A**). These include complete homoeologous triads derived from the A, B, and D subgenomes, specifically *TaCRY1a-6A/6B/6D*, *TaCRY1b-2A/2B/2D*, *TaCRY2-6A/6B/6D*, and *TaCRY3-7A/7B/7D*, which likely originated from allopolyploidization. Additionally, a homoeologous pair was identified between *TaUVR3-6A* and *TaUVR3-6D* (**Figure 4A**), which likely originated from allopolyploidization during wheat polyploidization rather than local duplication events. Furthermore, synteny comparison among multiple wheat species (**Figure 4B**), including diploid and tetraploid relatives, revealed that most *CRY/PHL* homoeologs have been retained in a subgenome specific manner following polyploidization, with occasional gene loss or rearrangement in specific subgenomes. Interspecific synteny analysis revealed no collinear *CRY/PHL* gene pairs between wheat and *Arabidopsis thaliana* (**Figure 4C**). In contrast, a total of 16 and 18 collinear gene pairs were identified between wheat and rice, and between wheat and maize, respectively (**Figure 4C**), indicating a high degree of syntenic conservation within the grass (*Poaceae*) lineage. While this pattern is consistent with lineage-specific evolution within grasses, the absence of collinearity with *Arabidopsis* alone does not definitively prove grass-specific expansion, as other factors such as differential gene loss or genome rearrangement may also contribute.

### 3.4 Physicochemical properties of TaCRY/PHL

To gain further insight into the physicochemical properties and potential functions of TaCRY/PHL, we analyzed the amino acid sequences of all identified TaCRY/PHL proteins (**Table 1**). The results showed that TaCRY/PHL proteins range from 545 to 712 amino acids in length, with corresponding molecular weights between 61.83 and 80.63 kDa. The predicted isoelectric points (pI) of TaCRY/PHL proteins spanned a wide range from 5.31 to 9.62, which distinctly segregated into two clusters. Nine members belonging to subgroups A and B possessed acidic pI values (around 5.5), while all five members of subgroup C and D exhibited basic pI values (around 9.0). All TaCRY/PHL proteins had instability indices greater than 40, suggesting that they are predicted to be unstable proteins. The grand average of hydropathicity (GRAVY) values were all negative, ranging from -0.491 to -0.328, which suggests that all TaCRY/PHL are predicted to be hydrophilic proteins. Subcellular localization predictions revealed a notable divergence correlated with subgroup affiliation. In subgroups A and B, all members except TaCRY1a-6B were predicted to localize to the nucleus or cytoplasm, whereas TaCRY1a-6B was predicted to be chloroplastic. In subgroup D, TaUVR3-6A/6D were predicted to localize to both chloroplasts or/and mitochondria, while the remaining three members in subgroup C were predicted to be exclusively chloroplast localized. These subcellular localization patterns imply a potential functional divergence of TaCRY/PHL subfamilies in plant growth, development, and stress responses.

**Table 1:** The characteristics and physicochemical properties of the proteins encoded by *CRY/PHL* genes in wheat.

Gene	Subgroups	Amino acids number	Molecular weight	Theoretical pI	Instability index	Aliphatic index	GRAVY	Subcellular localization prediction
*TaCRY1b-2A*	A	712	80626.7	5.54	53.09	76.03	-0.428	cytoplasm
*TaCRY1b-2B*	A	712	80497.49	5.41	54.06	76.43	-0.41	cytoplasm
*TaCRY1b-2D*	A	696	78841.74	5.58	52.95	77.08	-0.412	cytoplasm
*TaCRY1a-6D*	A	698	79049.55	5.52	52.02	74.63	-0.491	nucleus
*TaCRY1a-6B*	A	684	77644.21	5.55	51.61	74.61	-0.465	chloroplast
*TaCRY1a-6A*	A	698	79033.49	5.34	51.53	73.8	-0.475	nucleus
*TaCRY2-6B*	B	650	73258.94	5.62	41.17	85.49	-0.363	nucleus
*TaCRY2-6D*	B	653	73643.45	5.44	44.96	83.91	-0.351	nucleus
*TaCRY2-6A*	B	653	73450.14	5.31	43.87	85.25	-0.328	nucleus
*TaCRY3-7A*	C	593	65945.05	9.44	44.34	77.52	-0.394	chloroplast
*TaCRY3-7D*	C	597	66302.98	9.62	41.73	76.68	-0.376	chloroplast
*TaCRY3-7B*	C	598	66333.77	9.56	43.98	79.65	-0.346	chloroplast
*TaUVR3-6A*	D	555	62854.47	8.79	50.29	72.47	-0.484	chloroplast
*TaUVR3-6D*	D	545	61833.48	9.08	49.44	73.43	-0.475	chloroplast

### 3.5 Genetic structure, conserved motifs, and functional domains of TaCRY/PHL members

**Figure 5:**
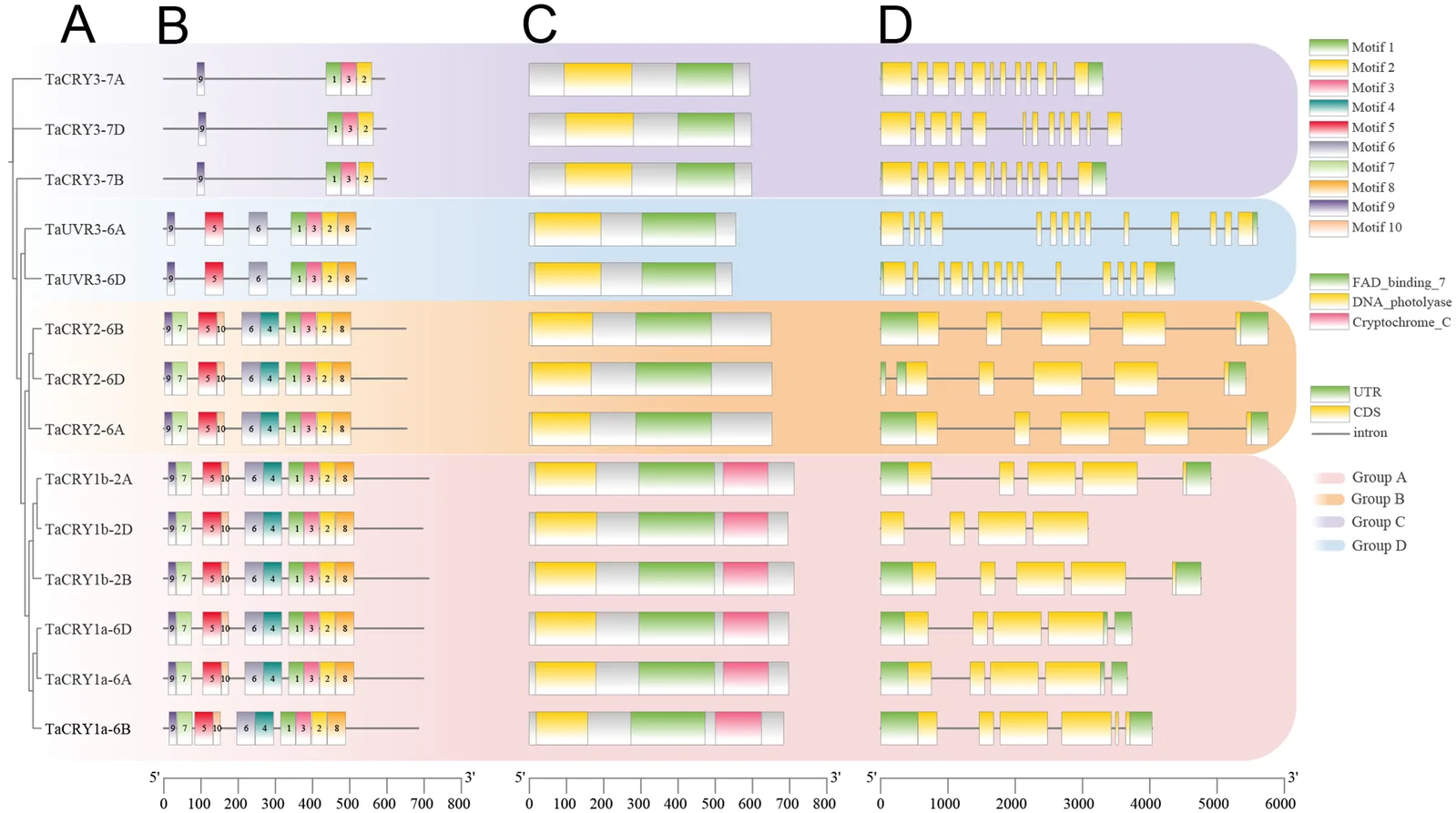
Analysis of conserved motifs, functional domains, and gene structures of the wheat CRY/PHL family. **(A)** Phylogenetic tree of CRY/PHL proteins in wheat. **(B)** Conserved motif distribution in TaCRY/PHL proteins. Ten motifs (Motifs 1-10) are indicated by differently colored boxes. **(C)** Conserved domain architecture of TaCRY/PHL proteins. The FAD-binding domain, the DNA photolyase, and Cryptochrome_C domain are shown by green, yellow, and pink boxes, respectively. **(D)** Exon-intron structures of *TaCRY/PHL* genes. Exons are represented by yellow boxes, introns by black lines, and untranslated regions (UTRs) by green boxes. The scale bar indicates the length of the genomic sequence (bp).

To systematically investigate the structural features and functional conservation of the *TaCRY/PHL* gene family, we analyzed their gene structures, conserved motifs, and functional domains (**Figure 5**). Conserved motif analysis revealed ten distinct motifs (Motifs 1-10) across the TaCRY/PHL protein sequences, with composition and arrangement varying among subgroups (**Figure 5B**). Notably, all members of subgroups A and B possessed the complete set of ten motifs, arranged in an identical order. In subgroup C, *TaCRY3-7A*, *TaCRY3-7B*, and *TaCRY3-7D* contained only Motifs 9, 1, 2 and 3, whereas *TaUVR3-6A* and *TaUVR3-6D* in subgroup D possessed Motifs 9, 5, 6, 1, 3, 8, 2 and 9. Among them, Motifs 9, 1, 2, and 3 were highly conserved across all *TaCRY/PHL* members, suggesting that they represent core and family-defining motifs corresponding to essential functional domains. Functional domain analysis revealed that all *TaCRY/PHL* members harbor two conserved domains, namely the FAD-binding domain and the DNA photolyase domain (**Figure 5C**). These two domains should together constitute the previously reported photolyase homology region (PHR), which is critical for blue light perception and signal transduction (Yan et al., 2016; Li et al., 2022). In addition, members of subgroup A also contained the Cryptochrome_C domain, which is known as the cryptochrome C-terminal extension (CCE) domain. Gene structure analysis revealed that members of subgroups A and B contained 4–6 exons and possessed both 5’and 3’-untranslated regions (UTRs), with the exception of *TaCRY1b-2D*, which lacked 5’-UTR (**Figure 5D**). Subgroup C and D members exhibited 12–14 exons, with *TaUVR3-6D*, *TaCRY3-7A*, and *TaCRY3-7B* having complete UTRs, *TaUVR3-6A* possessing only 3’-UTR, and *TaCRY3-7D* entirely lacking UTRs. Collectively, these results demonstrate that *TaCRY/PHL* members belonging to the same subgroup exhibit similar gene structures, motif compositions, and domain architectures, supporting the notion of functional conservation within each subgroup.

### 3.6 Cis-acting elements analysis of *TaCRY/PHL* promoters

**Figure 6:**
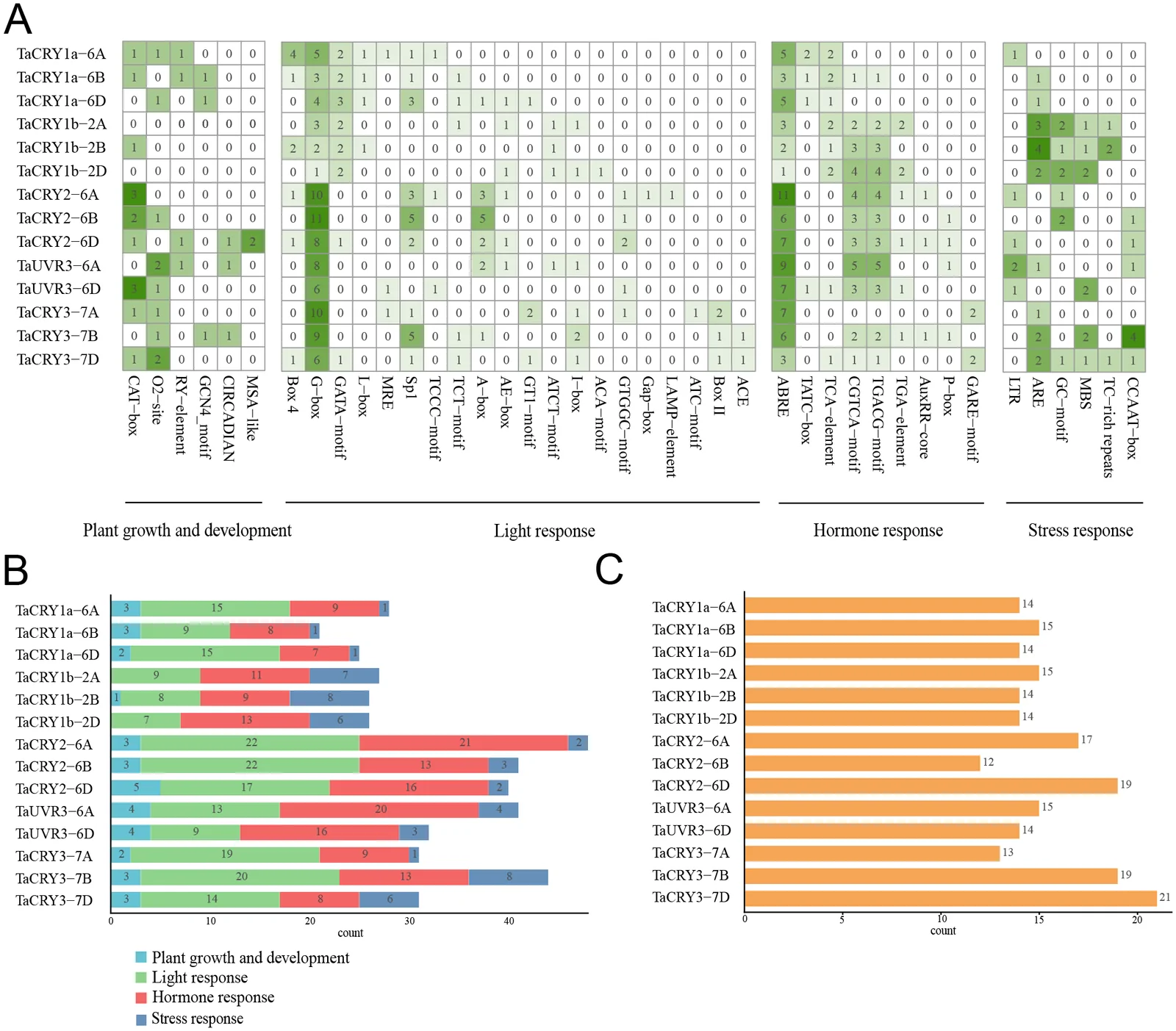
Cis-acting elements analysis of *TaCRY/PHLs* promoters. **(A)** The types and numbers of cis-acting elements in the promoters of *TaCRY/PHL* genes. **(B)** The number of cis-acting elements in each category.Different colors represent distinct categories. **(C)** The numbers of cis-acting element types in each *TaCRY/PHL* promoter.

To investigate the transcriptional regulation of the *TaCRY/PHL* family, we analyzed the promoter regions (2, 000 bp upstream of the translation start site) of each *TaCRY/PHL* members for cis-acting elements. A total of 41 distinct cis-acting elements were identified and categorized into four functional groups, including plant growth and development, light response, hormone response, and stress response (**Figure 6**). Among them, most *TaCRY/PHL* promoters contained a relatively high number of light-responsive elements (ranging from 7 to 22) and hormone-responsive elements (ranging from 7 to 21). The predominant light-responsive elements included G-box, Sp1, and A-box. Notably, *ELONGATED HYPOCOTYL5* (*HY5*), a key positive regulator of photomorphogenesis, has been reported to directly bind to the G-box cis-element in *TANDEM ZINC-FINGER/PLUS3* (*TZP*) promoter to activate its expression under far-red light (Xiao et al., 2022). Hormone-responsive elements were primarily represented by MeJA-responsive motifs (TGACG-motif and CGTCA-motif), salicylic acid-responsive elements (TCA-element), abscisic acid-responsive elements (ABRE), gibberellin-responsive elements (GARE-motif, TATC-box, and P-box), and auxin-responsive elements (TGA-element and AuxRR-core). Stress-responsive elements mainly included defense- and stress-related motifs (TC-rich repeats), drought-responsive elements (MBS), and low temperature-responsive elements (LTR). In addition, members of the *TaCRY3* subgroup harbored the greatest diversity of cis-regulatory elements, suggesting that this subgroup possesses a relatively complex potential transcriptional regulatory network among the wheat *CRY/PHL* family. These results suggest that the *TaCRY/PHL* family is may be potentially involved in hormone signaling, plant growth and development, and stress responses, thus suggesting their potential involvement in environmental adaptation in wheat, which warrants further experimental validation.

### 3.7 Tissue-specific expression patterns of *TaCRY/PHL* genes

**Figure 7:**
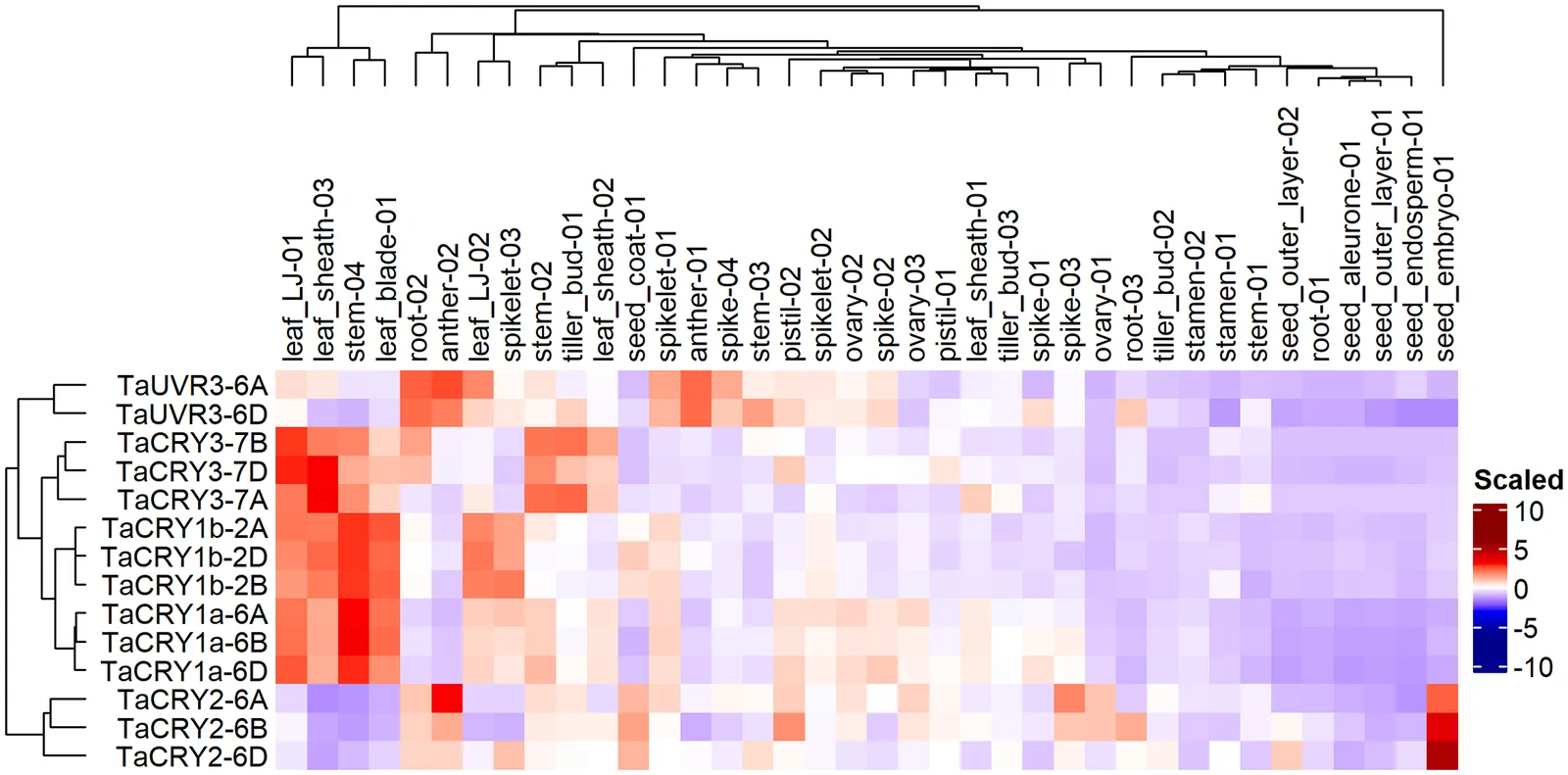
Tissue-specific expression patterns of *TaCRY/PHL* genes in wheat. RNA-seq data were obtained from the NCBI SRA database. Expression levels are shown as FPKM values row-scaled using the scale() function in R, with the color scale from blue (low expression) to red (high expression). Tissues analyzed include root, stem, leaf blade, leaf sheath, leaf LJ, tiller bud, anther, pistil, ovary, seed coat, and spike.

To elucidate the potential biological functions of *TaCRY/PHL* genes during wheat growth and development, tissue-specific expression patterns were analyzed using publicly available RNA-seq data from the NCBI Sequence Read Archive (SRA). The expression profiles of *TaCRY/PHL* genes across various tissues, including root, stem, leaf blade, leaf sheath, leaf LJ, tiller bud, anther, pistil, ovary, seed coat, and spike, are presented in **Figure 7**. The results showed that members of subgroups A and C, including *TaCRY1a-6A/6B/6D*, *TaCRY1b-2A/2B/2D*, and *TaCRY3-7A/7B/7D*, exhibited predominant expression in vegetative tissues such as leaf lamina joint (LJ), leaf sheath, stem, and leaf blade. In contrast, *TaUVR3-6A* and *TaUVR3-6D* exhibited highly specific expression patterns, with transcripts predominantly detected in root, anther, spikelet, and spike tissues. Members of the subgroup B (*TaCRY2-6A/6B/6D*) were highly expressed in anther, seed coat, pistil, spike, and seed embryo. Collectively, these results suggest functional divergence among different *TaCRY/PHL* subgroups, with subgroup A and C members mainly associated with vegetative growth, while subgroup B and D members may contribute to both reproductive organ development.

### 3.8 Expression patterns of *TaCRY/PHL* genes during LJ development

**Figure 8:**
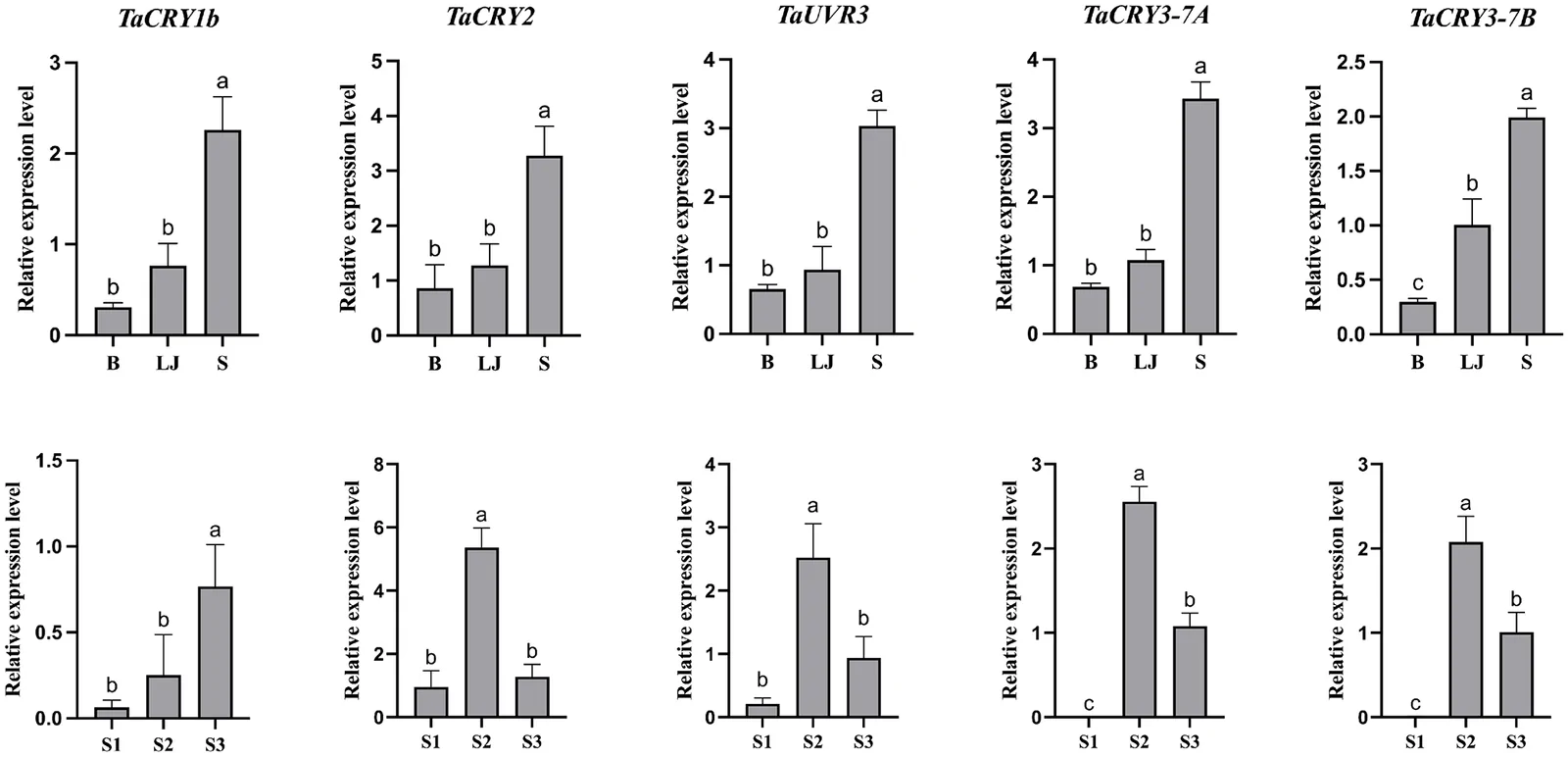
Expression analysis of *TaCRY/PHL* genes during leaf angle formation by qRT-PCR. B, LJ, and S denote leaf blade, leaf lamina joint, and leaf sheath, respectively. S1, S2, and S3 represent the three developmental stages of the leaf lamina joint, corresponding to 4, 8, and 12 days after emergence, respectively. Error bars represent the standard deviation (SD) of three biological replicates. Different lowercase letters above the bars indicate significant differences among samples or developmental stages at *p* < 0.05.

The presence and development of the lamina joint (LJ) are critical determinants of leaf angle size (Wang et al., 2020; Wang et al., 2021). To investigate the potential functions of *TaCRY/PHL* genes in leaf angle formation and LJ development, we examined the expression patterns of five *TaCRY* genes from four subgroups in different leaf tissues (leaf blade, leaf sheath, and LJ) across three LJ developmental stages (S1, S2, and S3) using qRT-PCR (**Figure 8**). The results showed that *TaCRY1b*, *TaCRY2*, *TaUVR3*, *TaCRY3-7A*, and *TaCRY3-7B* were expressed at higher levels in leaf sheath and LJ than that in blade. During LJ development, the expression of *TaCRY1b* increased progressively, reaching its maximum at stage S3. In contrast, although the expression levels of *TaCRY2*, *TaUVR3*, *TaCRY3-7A*, and *TaCRY3-7B* also showed an increasing trend, they peaked at stage S2 and subsequently declined. In summary, these distinct expression patterns suggest that *TaCRY/PHL* members may play stage-specific roles in LJ development.

### 3.9 Subcellular localization of TaCRY1b and its interaction with TaLG2/TaLG2L

**Figure 9:**
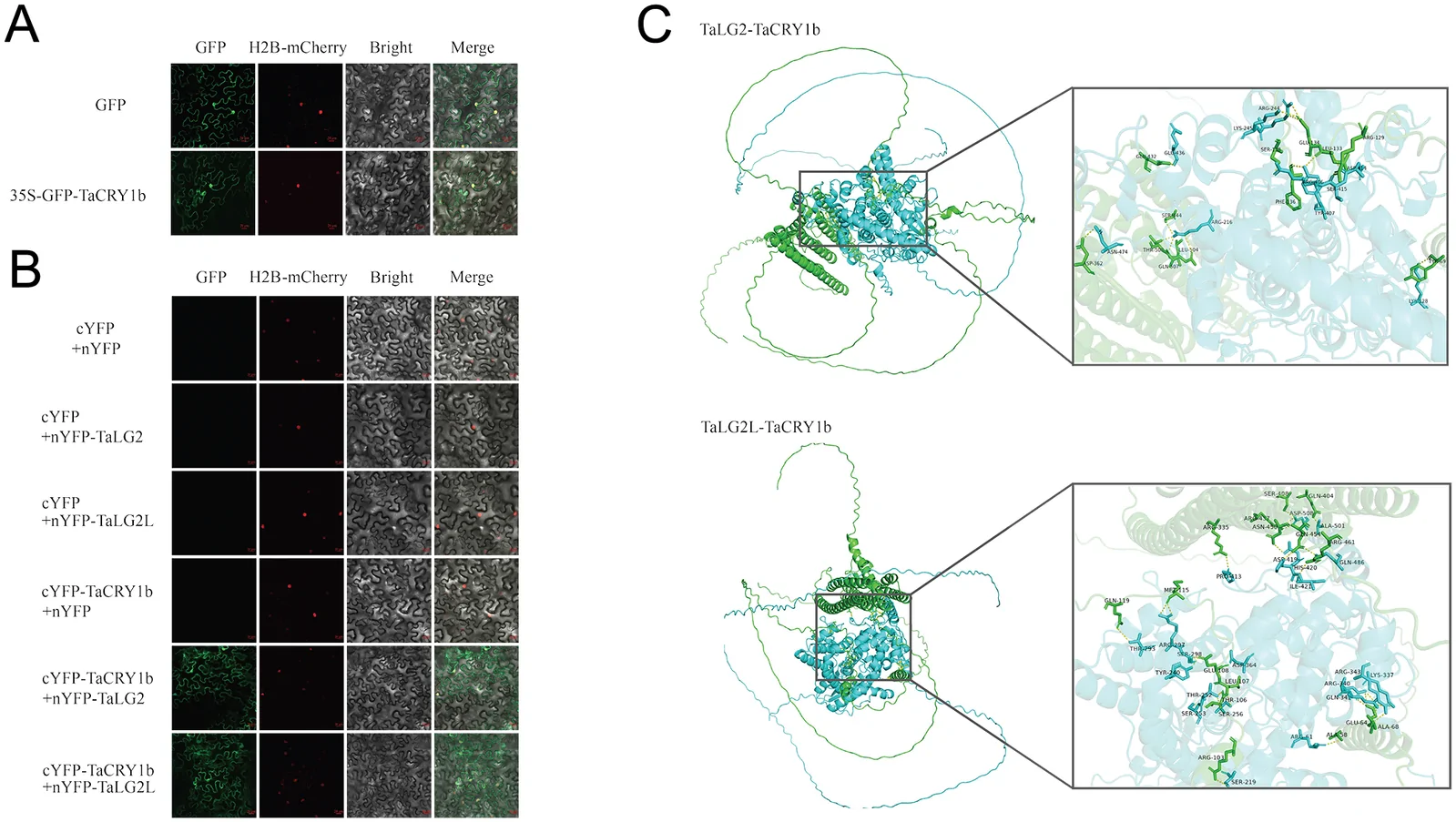
Subcellular localization of TaCRY1b and validation of its interaction with TaLG2/TaLG2L. **(A, B)** Subcellular localization of TaCRY1b and verification of the interaction analysis between TaCRY1b and TaLG2/TaLG2L in plant by BiFC **(B)**. GFP or YFP fluorescence (green) co-localizes with the nuclear marker H2B-mCherry (red) in tobacco epidermal cells. Bar = 20μm. **(C)** The predicted interaction interface between TaLG2/TaLG2L and TaCRY1b through AlphaFold3. Predicted interaction residues are highlighted. Detailed residue pairs are listed in the **Supplementary Table 4**.

Previous yeast two-hybrid (Y2H) screening in our laboratory identified TaCRY1b as a candidate interacting protein of TaLG2/TaLG2L (Wang et al., 2026), which are key regulators of leaf lamina joint development in wheat. To further investigate the subcellular localization of TaCRY1b and validate its interaction with TaLG2/TaLG2L in plant cells, we performed transient expression assays in tobacco epidermal cells. The results showed that the 35S-GFP-TaCRY1b fusion protein localized to both the cytoplasm and the nucleus, with its green fluorescent signals overlapping the H2B-mCherry nuclear marker (**Figure 9A**). Previous Y2H results showed that TaCRY1b interacts with TaLG2 and TaLG2L (Wang et al., 2026). In this study, bimolecular fluorescence complementation (BiFC) assays further confirmed that this interaction occurs in both cytoplasm and the nucleus (**Figure 9B**). To further dissect the molecular basis of the interaction between TaCRY1b and TaLG2/TaLG2L, we performed protein-protein interaction prediction using AlphaFold3 (**Figure 9C**).The prediction suggested a putative contact interface between TaLG2/TaLG2L and TaCRY1b, involving multiple complementary residue pairs primarily through polar contacts with a relative low confidence metrics (**Supplementary Figure 1**; **Supplementary Table 4**). Notably, most of the TaCRY1b residues predicted to participate in these interactions are located within the FAD-binding domain and the DNA photolyase domain, which together constitute the PHR domain. Overall, these findings identify TaCRY1b as an interacting protein of TaLG2/TaLG2L, yet the precise binding sites and its functional contribution to leaf angle development need additional experimental evidence.

## 4 Discussion

Cryptochromes function as conserved blue light/UV-A photoreceptors across eukaryotes and govern diverse developmental and environmental adaptation processes in higher plants. In this study, we systematically identified 57 *CRY/PHL* genes across five wheat species, rice, maize, and *Arabidopsis* thaliana, including 14 members in hexaploid common wheat. Phylogenetic analysis resolved the wheat CRY/PHLs into four distinct subfamilies, corroborating previous reports on the evolutionary divergence of plant cryptochromes (Cao et al., 2020; Lu et al., 2024). All TaCRY/PHL proteins contain the conserved PHR domain, while only TaCRY1 and TaCRY2 possess the CCT, indicating functional divergence among subfamilies. A previous genome-wide survey identified 12 *TaCRY* genes in wheat using three HMM models (PF00875, PF03441, and PF12546) (Cui et al., 2025). Our two-domain strategy (PF00875 and PF03441) identified the same 12 members plus two additional UVR3 genes (TaUVR3-6A and TaUVR3-6D). The absence of these two UVR3 members in the earlier report is likely due to incomplete annotation or sequence divergence of the PF12546 domain. The results in this study provides a more comprehensive view of the wheat cryptochrome/photolyase superfamily. Synteny analysis revealed extensive collinearity among wheat, rice and maize but not with *Arabidopsis*, consistent with lineage-specific evolution within the grass family. Promoter cis-element analysis showed that *TaCRY/PHL* promoters contain multiple predicted light-, hormone- and stress-responsive elements. Expression profiling demonstrated that *TaCRY1s* and *TaCRY3s* are predominantly expressed in vegetative tissues, whereas *TaCRY2s* and *TaUVR3s* are more abundant in reproductive organs. Subcellular localization showed that TaCRY1b localized to both the cytoplasm and nucleus. Subsequent, Y2H and BiFC assay further verified its physical interactions with TaLG2 and TaLG2L. Nevertheless, these findings still require corroboration via co-immunoprecipitation (Co-IP) assays performed in wheat. Furthermore, the key residues within the PHR domain of TaCRY1b that mediate this interaction were predicted by AlphaFold3. Taken together, our results decipher the evolutionary characteristics of wheat CRY/photolyase family members and pinpoint TaCRY1b as a key interactor of TaLG2/TaLG2L, offering valuable clues for further mechanistic research on leaf angle regulation.

The evolutionary history of the CRY/PHL family in plants has been a subject of extensive investigation. In this study, the phylogenetic tree, covering 73 green plant species, showed that CRY/PHL proteins in the Triticeae group are mainly confined to four major clades (clade02, clade05, clade10 and clade12), corresponding to CRY3, UVR3, CRY2 and CRY1, respectively. This distribution is in agreement with recent large-scale evolutionary analyses showing that *CRY2* originated from a common ancestor of land plants (embryophytes), whereas *CRY1* arose from a common ancestor of seed plants (Lu et al., 2024). In addition, we detected extensive syntenic blocks among wheat, rice and maize *CRY/PHL* genes, but no collinearity between wheat and *Arabidopsis*, suggesting that the *CRY/PHL* family may have undergone lineage-specific expansion in grasses, potentially driven by polyploidisation and segmental duplication, although other mechanisms cannot be excluded (Chaves et al., 2011). Conserved domain ananlysis found that all TaCRY/PHL proteins contain the PHR domain, which is essential for FAD binding and blue light perception, but the CCE (CCT) domain is present only in TaCRY1 and TaCRY2. Upon photoactivation, CRY/PHL proteins trigger a cascade of molecular events including tetramerization, photobody biogenesis and physical association with downstream signaling partners for subsequent blue-light signal transduction (Wang and Lin, 2020). Studies have demonstrated that the PHR domain, an evolutionarily conserved core domain derived from ancestral photolyases in cryptochromes, mainly functions in flavin cofactor binding, light signal perception and protein-protein interaction, serving as the common structural scaffold supporting the biochemical activities of all cryptochromes (Yang et al., 2000; Parico et al., 2020; DeOliveira and Crane, 2024). As an evolutionarily acquired signal-output module, the CCT domain acts as a pivotal functional element driving the evolutionary transition of cryptochromes from DNA-repairing photolyases into blue-light photoreceptors (Yang et al; DeOliveira and Crane, 2024). For example, the C-terminal extension of CRY2 as well as its phosphorylation is indispensable to sustain the liquid-like property of CRY2 photobodies (Wang et al., 2021). Accordingly, it is hypothesized that wheat CRY1 and CRY2 transduce blue-light signals via intramolecular PHR-CCT interactions. Upon blue-light irradiation, the PHR domain perceives light signals and undergoes redox modification to release the intramolecular repression imposed on CCT under darkness (Wang and Lin, 2020). The liberated CCT further initiates downstream regulatory cascades governing photomorphogenesis (Jiang et al., 2025), circadian rhythm (Shi et al., 2026) and photoperiodic flowering (Liu et al., 2008; Zhang et al., 2008). In contrast to CRY1, the typical C-terminal extension (CCT) was absent in TaCRY2, TaCRY3 and TaUVR3. This structural difference implies that they may have diverged in function, potentially acting in DNA repair rather than canonical blue-light signaling. Such structural divergence may lead to functional differentiation among the four wheat cryptochrome members, which is consistent with the evolutionary trajectory from primitive photolyases to canonical blue-light signaling cryptochromes observed in other plant species.

The tissue-specific expression patterns of the *TaCRY/PHL* subfamilies clearly indicate functional divergence. In this study, *TaCRY1s* and *TaCRY3s* are highly expressed in vegetative organs, whereas *TaCRY2s* and *TaUVR3s* are mainly expressed in reproductive tissues such as anthers, pistils and seeds. Similar patterns have been observed in maize, where *ZmCRY1a1* and *ZmCRY1a2* show high expression level in leaf (Fan et al., 2023), and in tea, where *CsCRY1/2* are higher expressed in leaves and roots (Tang et al., 2020), suggesting that the involvement of CRY1 in vegetative growth is a relatively conserved trait among angiosperms. According to the current model, plant cryptochromes exist as inactive monomers in the dark; absorption of blue light triggers photoreduction of the flavin adenine dinucleotide (FAD) chromophore, leading to conformational changes and homooligomerization (Shao et al., 2020; Wang and Lin, 2020). In line with this, the promoters of *TaCRY/PHL* genes contain multiple predicted light-responsive (G-box) and hormone-responsive (ABRE, TGACG-motif) cis-elements, indicating tight integration with both environmental light signals and endogenous hormonal cues. Importantly, during three successive stages of LJ development, *TaCRY1b* expression gradually increased and peaked at the late stage (S3), whereas other *TaCRY/PHL* members peaked earlier (S2). This unique temporal profile suggests that TaCRY1b may be associated with the later stages of LJ development. The presence of multiple predicted light- and hormone-responsive cis-elements (G-box, ABRE, TGACG-motif) in the *TaCRY/PHL* promoters suggests a potential link between *TaCRY1b* expression and light/hormonal signals. In contrast, the chloroplast-targeted *TaCRY3*s subfamily is likely to sense light intensity and redox status directly within plastids. It may harness plastidial retrograde modules to maintain photosynthetic efficiency under stress conditions, a process that could be critical for seed vigor and adaptation to high-light environments (Chaves et al., 2011; Lee and Kim, 2024).

Leaf angle is a key determinant of plant architecture and canopy light interception (Wang et al., 2026). In maize, *LIGULELESS1* (*LG1*) and *LIGULELESS2* (*LG2*) directly regulates leaf angle by controlling ligule and auricle development (Becraft et al., 1990; Walsh and Freeling, 1999). In rice, homologs *OsLG2* and *OsLG2L* play analogous roles in lamina joint formation (Wang et al., 2021). In addition, plant hormones and light signals also play important regulatory roles in LJ development and leaf angle formation (Xu et al., 2021; Wang et al., 2026). Recently, it was shown that under high R/FR, PhyB stabilizes LIGULELESS 1 (LG1), which in turn inhibits *HB53* (a homeobox TF) to suppress cell elongation (Shi et al., 2024). Notably, blue light is an effective type of light for regulating leaf angle, as it upregulates mRNA levels of several cytochrome P450 enzymes including *CYP85A1*, thereby promoting castasterone synthesis and subsequently accelerating rice lamina bending and unrolling (Asahina et al., 2014). However, it remains unknown whether blue light or its photoreceptors play a regulatory role in wheat LJ development and leaf angle formation. In this study, the key regulators of LJ initiation, TaLG2/TaLG2L, were confirmed to interact with TaCRY1b in both the cytoplasm and the nucleus (**Figure 9B**). Moreover, AlphaFold3 further predicted the interaction interface, revealing that it involves multiple amino acid residues within the PHR domain of TaCRY1b (**Figure 9C**). This is consistent with the established view that the PHR domain of plant cryptochromes acts as a central platform for protein-protein interactions (Wang and Lin, 2020; Sarkar et al., 2025). In *Arabidopsis*, the C-terminal domain of CRY1 (CCT1) is maintained in an autoinhibited state in the dark. Upon blue light, the PHR domain undergoes a redox reaction that relieves this repression, allowing the CCT domain to interact with downstream signaling partners such as COP1 (Yang et al; Parico et al., 2020). Whether a similar mechanism operates in wheat, and whether the TaCRY1b-TaLG2/TaLG2L module is modulated by blue light, remain to be determined. Recent advances have shown that photoactivated CRY/PHLs control gene expression via both the classical light-induced fit (lock-and-key) mechanism and light-induced liquid-liquid phase separation (LLPS), the latter serving to concentrate CRY-interacting proteins and their targets into nuclear photobodies, thus accelerating biochemical reaction rates (Wang and Lin, 2025). For example, CRY2-mediated LLPS with the mRNA m6A methyltransferase complex (*MTA*/*MTB*/*FIP37*) and with the splicing factor *MAC3A*/*B* controls epitranscriptome and alternative splicing in response to blue light (Wang et al., 2021; Jiang et al., 2023). Whether TaCRY1b undergoes similar LLPS with TaLG2/TaLG2L remains to be investigated. Notably, the expression of *TaCRY1b* gradually increases during LJ development, suggesting its potential involvement in the later stages of LJ differentiation. Interestingly, positive selection on *CRY1/2* genes and convergent loss of the VP motif in CRY2 have been reported in aquatic angiosperms as adaptations to underwater light conditions (Lu et al., 2024). Despite wheat being a land plant, numerous light-responsive elements were identified in the *TaCRY1b* promoter, and its fluctuating expression during lamina joint development indicates light-mediated transcriptional control of *TaCRY1b*, which remains to be further characterized.

## Data Availability

The datasets presented in this study can be found in online repositories. The names of the repository/repositories and accession number(s) can be found in the article/**Supplementary Material**.
